# Factors associated with changes in endothelin-1 gene expression in patients with diabetic retinopathy in type 2 diabetes mellitus

**Published:** 2010-07-10

**Authors:** Barbara Strzalka-Mrozik, Agnieszka Nowak, Joanna Gola, Malgorzata Kowalczyk, Malgorzata Kapral, Urszula Mazurek

**Affiliations:** 1 Department of Molecular Biology, Medical University of Silesia, Sosnowiec, Poland; 2 Department of Ophthalmology, Medical University of Silesia, Sosnowiec, Poland; 3 Department of Medical Genetics, Medical University of Silesia, Sosnowiec, Poland; 4 Department of Biochemistry, Medical University of Silesia, Sosnowiec, Poland

## Abstract

**Purpose:**

To (i) investigate expression of the endothelin-1 (*ET-1*) gene in peripheral blood mononuclear cells (PBMCs) of patients with type 2 diabetes mellitus (DM) and (ii) determine what correlations, if any, exist between expression of *ET-1* in patients with type 2 DM and treatment, clinical features, and biochemical markers in diabetic retinopathy (DR).

**Methods:**

The study group included 58 patients with type 2 DM, subdivided into three subgroups: those without DR (n=19), those with nonproliferative DR (NPDR; n=28), and those with proliferative DR (PDR; n=11). The control group consisted of 60 individuals. In all groups the mRNA level of *ET-1* was estimated using real-time quantitative reverse transcription PCR.

**Results:**

The mRNA level of *ET-1* in patients with NPDR was significantly higher than in those without DR. An increase in *ET-1* expression was observed in patients with PDR as opposed to those without DR. Compared to controls, the mRNA level of *ET-1* was significantly higher both in patients with NPDR and those with PDR. Duration of DM, insulin therapy, and serum creatinine levels were associated with increased mRNA level of *ET-1*, whereas medication with sulfonylurea or angiotensin-converting enzyme inhibitors had the opposite effect.

**Conclusions:**

Expression of *ET-1* in PBMCs may be associated with severity of DR in patients with type 2 DM. Long-standing clinical course of DR; medication with insulin, sulfonylurea, or ACE inhibitors; and serum creatinine levels are factors possibly associated with changes in *ET-1* expression in PBMCs.

## Introduction

Alterations in activity of the endothelin (ET) system are believed to underlie development of structural and functional lesions related to type 2 diabetes mellitus (DM). Plasma levels of endothelins — produced and secreted mostly by epithelial cells—change during the course of type 2 DM. Such variations are generally attributed to an increased level of the endothelin-1 (ET-1) protein, which is associated with severity of endothelial cell injury [1].

Under normal physiologic conditions, ET-1 is a crucial protein for the non-neuronal autoregulation of retinal blood flow. It not only causes constriction and increased tonicity of blood vessels but also enhances endothelial production of such vasodilators as nitric oxide and prostacyclines [2]. Further, ET-1 is involved in stimulating proliferation and migration of endothelial cells and has potential mitogenic activity over smooth muscle cells [3].

The relationship between ET-1 plasma concentration and degree of progression of diabetic retinopathy (DR) has been demonstrated in many studies [2,4–8]. In the eye, local production of ET-1 may result from retinal disease [4]. On the other hand, the level of ET-1 in the vitreous may result, in part, from damage to the blood–retina barrier and penetrable vessels. Diabetic microangiopathy occurs not only in ocular tissues but also in the kidney, nervous system, and skin—suggesting etiogenic mechanisms not specific to the organ in question [9,10]. ET-1 is produced by endothelial cells, vascular smooth muscle cells, macrophages, leucocytes, cardiomyocytes, and fibroblasts [10]. Since leucocytes produce ET-1, peripheral blood mononuclear cells (PBMCs) are a potential source of this protein, which may influence the structural and functional microcirculatory changes observed in DM. There are, however, no published data regarding the mRNA level of the *ET-1* gene in PBMCs, as measured by real-time quantitative reverse transcription PCR (qRT–PCR), in patients with type 2 DM. Furthermore, possible correlations between the mRNA level of the *ET-1* gene in PBMCs and relevant clinical features or biochemical parameters have not yet been described.

The goals of this study were to investigate the mRNA level of *ET-1* in PBMCs of patients affected with type 2 DM, with or without concomitant DR, and examine possible correlations between expression of *ET-1* within this group of patients and treatment, clinical features, and biochemical markers of DR.

## Methods

### Patients

Included in this study were 58 patients with type 2 DM treated in the Department of Ophthalmology, Medical University of Silesia, St. Barbara Hospital. All patients were informed about the research and signed the informed consent. The study was approved by the Bioethics Committee of Medical University of Silesia, Katowice (decision NN 6501/146/I/05). Diagnosis of type 2 DM was based on World Health Organization criteria [11]. The clinical features and biochemical markers are presented in Table 1. Patients with type 2 DM were further subdivided into three groups: those without DR (n=19), comprising six males and 13 females, mean age=63.4 years (range 49–79 years); those with nonproliferative diabetic retinopathy (NPDR; n=28), comprising 12 males and 16 females, mean age=58.5 years (range 42–83 years); and those with proliferative diabetic retinopathy (PDR; n=11), comprising seven males and four females, mean age=67 years (range 52–75 years). No patient with type 2 DM without DR was diagnosed with diabetic macular edema. The presence of diabetic macular edema was revealed in patients with DR. The control group consisted of 60 individuals, all with a negative history of DM, normal fasting serum glucose, and normal findings on ophthalmoscopic examination (Table 1). Exclusion criteria were as follows: past or active retinal or optic nerve disorders, primary open-angle glaucoma, angina pectoris, hypertrophic cardiomyopathy, history of myocardial infarction, stroke, peripheral vascular diseases, diabetic nephropathy, neuropathy, or microalbuminuria as confirmed by a strip test (Micral Test II, Roche, Germany) of a morning urine sample. There were no statistically significant differences between the study groups and the control group with regard to gender, age, or body mass index. In DM patients, arterial hypertension was more frequent (Fisher’s exact test, p=0.005) and glucose concentrations were higher (Mann–Whitney U test, p<0.001) than in control group individuals.

**Table 1:** Selected clinical features and biochemical markers of type 2 diabetus mellitus (DM) patients and controls

**Characteristic**	**Type 2 DM (n=58)**	**Controls (n=60)**
**Gender**		
M	21	33
F	37	27
**Age (year)**	60.0 (42.0–83.0)	61.0 (52.0–76.0)
**BMI (kg/m2)**	29.4 (22.0–44.0)	27.0 (21.0–32.0)
**BMI**		
Normal	9	24
Overweight	23	24
Obese	26	12
**Smoking**		
Yes	6	0
No	52	60
**Arterial hypertension**		
Yes	40	4
No	18	56
*Glycemia	142.5 (69.0–387.0)	100.0 (89.0–108.0)
*Serum creatinine	0.9 (0.5–1.1)	1.2 (0.9–1.1)
*Total cholesterol	193.0 (139.0–326.0)	220.0 (184.0–267.0)
*Triglycerides	158.0 (69.0–343.0)	110.0 (86.0–193.0)
*HDL cholesterol	49.5 (40.0–64.0)	65.0 (47.0–128.0)
*LDL cholesterol	125.5 (100.0–20.0)	147.0 (52.0–199.0)
**Glycosylated hemoglobin (%)**	7.5 (5.3–9.8)	-
**Length of clinical course (year)**	10.5 (1.0–29.0)	-
**Type of retinopathy**		
No DR	19	-
NPDR	28	-
PDR	11	-
**Diabetic macular edema**		
Yes	0	-
No	58	-
**Frequency of glycemic control**		
Less than once daily	14	-
Once daily	14	-
Twice daily	13	-
Thrice daily	17	-
**Insulin therapy**		
Yes	31	-
No	27	-
**Length of insulin therapy (years)**	7.0 (0–25.0)	-
**Medication with oral anti-diabetic drugs**		
Yes	44	-
No	14	-
**Type of the anti-diabetic drug**		
Metformin	36	-
Sulfonylureas	24	-
Alpha-glucosidase inhibitor	6	-
**Type of anti-hypertensive drug**		
β-blocker	13	-
Ca-blocker	11	-
ACE inhibitor	29	-
Diuretics	12	-

Values of clinical and biochemical parameters are expressed as median (minimum–maximum). Abbreviations: M represents male; F represents female. *Expressed as mg%.

### RNA extraction from peripheral blood mononuclear cells

Venous blood samples were collected into EDTA tubes, and a 7.5-ml sample from each patient was centrifuged on a Ficoll-Conray gradient (specific gravity 1.077; Immunobiological Co., Gumma, Japan) immediately after blood collection.

Total RNA was extracted from specimens using a commercially available kit (Total RNA Prep Plus kit; A&A Biotechnology, Gdansk, Poland), based on the Chomczynski and Sacchi method, and according to manufacturer's instructions [12]. RNA extracts were treated with DNase I (MBI Fermentas, Vilnius, Lithuania), according to manufacturer's instructions. The quality of extracts was checked electrophoretically using 0.8% agarose gel stained with ethidium bromide. Results were analyzed and recorded using the gel documentation system 1D Bas-Sys (Biotech-Fisher, Perth, Australia). Total RNA concentration was determined by spectrophotometric measurement at A260, A280, and A310 in 5-μl capillary tubes, using the Gene Quant II RNA/DNA Calculator (Pharmacia Biotech, Cambridge, UK).

### Real-time quantitative reverse transcription PCR assay

The mRNA level of *ET-1* and glyceraldehyde-3-phosphate dehydrogenase (*GAPDH*) genes were evaluated on the basis of mRNA copy number per 1 μg of total RNA, using real-time qRT–PCR and SYBR Green I chemistry (SYBR Green Quantitect RT–PCR kit; QIAGEN, Valencia, CA). Analysis was performed using an Opticon™ DNA Engine Continuous Fluorescence Detector (MJ Research, Watertown, MA). qRT–PCR assays were performed in triplicate for all 81 samples. *GAPDH* was included to monitor RT–PCR efficiency for all samples. Oligonucleotide primers specific for: *ET-1* (5′-CCA ATC TTG GAA CAG TCT TTT CCT-3′ forward, 5′-GGA CAT CAT TTG GGT CAA CAC TCC-3′ reverse) and *GAPDH* (5′-GAA GGT GAA GGT CGG AGT-3′ forward, 5′-GAA GAT GGT GAT GGG ATT C-3′ reverse) genes were described previously by Berger et al. [13] and Ercolani et al. [14], respectively.

The thermal profile for one-step RT–PCR was as follows: 50 °C for 30 min for reverse transcription, 95 °C for 15 min, 40 cycles at 94 °C for 45 s, 54 °C for 45 s, and 72 °C for 35 s. The point at which the PCR product is first detected above a fixed threshold— termed cycle threshold (C_t_)—was determined for each sample, and the average C_t_ of triplicate samples was calculated. Each run was completed using melting curve analysis to confirm specificity of the amplification and absence of primer dimers. RT–PCR products were separated on 6% polyacrylamide gels and visualized with silver salts.

To quantify the results obtained by RT–PCR for *ET-1* and *GAPDH*, the standard curve method was used [15,16]. To simultaneously detect the expression profile of each investigated gene, commercially available standards of β-actin (*ACTB*) cDNA (TaqMan^®^ DNA Template Reagent kit; PE Applied Biosystems, Inc., Foster, CA) were used at five different concentrations (ranging from 400 to 8,000 copies of *ACTB* cDNA).

Amplification plots for each dilution of the commercially available standard template were used to determine C_t_ values [15,16]. Correlation coefficients for standard curves ranged from 0.988 to 0.995, indicating a high degree of confidence for measurement of the copy number of molecules in each sample. Copy numbers of analyzed mRNAs were calculated from the linear regression of the standard curve.

### Statistical analyses

Statistical analyses were performed using Statistica 8.0 software (StatSoft, Tulsa, OK), with a significance level set at p<0.05. Values are expressed as median (Me), minimum and maximum, and lower (25th) and upper (75th) quartiles. The hypertension prevalence rate was tested with Fisher's exact test. For statistically testing outlying values in continuous variables, Dixon’s test was used. For the relationship between DR and *ET-1*, the mRNA level was tested using Kruskal–Wallis and post hoc paired comparisons based on mean ranks. Differences in individual qualitative variables and expression of *ET-1* between analyzed groups were tested using the Mann–Whitney *U*-test. The relationship between quantitative variables and expression of the *ET-1* gene was determined by Spearman’s rank correlation test.

## Results

In this study, the mRNA level of the *ET-1* gene in PBMCs from patients with type 2 DM complicated by retinopathy was determined using real-time qRT–PCR. Risk factors, with possible influence on mRNA levels of the gene in this group of patients, were also defined.

In the first stage of the study, specificity of the RT–PCR assay for the target genes was confirmed experimentally by PAGE and amplimer melting temperature. For each RT–PCR product, a single peak was obtained by melting curve analysis at the expected temperatures: *ET-1*, 78.8 °C ±0.16; *GAPDH*, 80.1 °C ±0.22 (data not shown). Gel electrophoresis revealed the presence of a single product of the predicted length (*ET-1*, 269 bp; *GAPDH*, 226 bp; data not shown).

**Figure 1:**
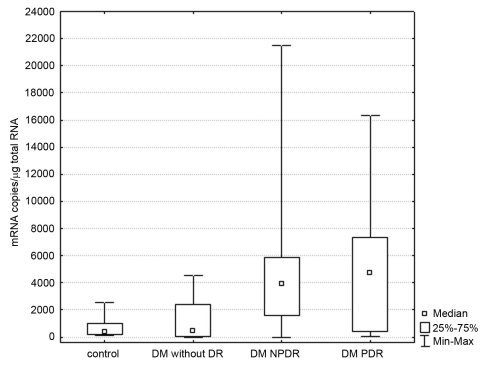
Comparison of number of copies of endothelin-1 (ET-1) mRNA in patients and controls. Statistical significance was found for control versus nonproliferative diabetic retinopathy (NPDR; p=0.036), control versus proliferative diabetic retinopathy (PDR; p=0.026), diabetes mellitus (DM) without DR versus NPDR (p=0.005), and DM without DR versus PDR (p=0.007). Boxes and whisker plots present medians±quartiles and minimum-maximum values of copy number per 1 μg total RNA.

In the second stage of the study, levels of ET-1 mRNA in patients and controls were assessed (Figure 1). Among patients with type 2 DM, the greatest number of ET-1 mRNA copies/μg total RNA was found in the PDR subgroup (Me=4745.0), somewhat fewer in the NPDR subgroup (Me=3893.1), and the least in DM patients without DR (Me=487.8). In the control group, there were fewer copies of ET-1 mRNA/μg total RNA than in patients with type 2 DM (Me=431.0). Statistical analyses revealed differences between analyzed groups (p=0.0008, Kruskal–Wallis=16.81). The ET-1 mRNA level in PDR and NPDR patients was significantly higher than in diabetic patients without DR (post hoc paired comparisons based on mean ranks, p=0.007 and p=0.005, respectively).

No statistically significant difference in the number of copies of ET-1/μg total RNA was found between NPDR and PDR patients. The mRNA level of ET-1 was significantly higher in the NPDR and PDR groups (post hoc paired comparisons based on mean ranks, p=0.036 and p=0.026, respectively) compared to controls. No statistically significant difference in the mRNA level of ET-1 was found between DM patients without DR and controls.

In the third stage of the study, correlations between expression of *ET-1* in patients with type 2 DM and treatment, clinical features, and biochemical markers of DR were investigated (Table 2 and Table 3). Among all analyzed nonparametric variables, only insulin therapy and medication with sulfonylurea or angiotensin-converting enzyme (ACE) inhibitors had any influence on expression levels of *ET-1*. Patients treated with insulin showed increased expression of the gene (Mann–Whitney *U* test, p=0.003), whereas the mRNA level was significantly lower in patients receiving sulfonylurea medication (Mann–Whitney *U* test, p=0.004) or ACE inhibitors (Mann–Whitney *U* test, p=0.029). Parametric variables associated with changes in the expression of *ET-1* included prolonged clinical course of DM and increased creatinine levels in venous blood. Both the long-standing clinical course of DM (Spearman test, p=0.013, r=0.292) and increased levels of serum creatinine (Spearman test, p=0.023, r=0.368) correlated with increased mRNA level of *ET-1*.

**Table 2:** Factors associated with changes in endothelin (*ET*)-1 gene expression in patients with type 2 diabetus mellitus (DM)

**Variable**	**n**	***ET-1* mRNA (copies/mg total RNA)^a^**	**p**
**Gender**			
M	21	2620 (267–4383)	NS
F	37	3385 (563–5242)	
**Smoking**			
Yes	6	4052 (1382–6035)	NS
No	52	2620 (283–4549)	
**Insulin therapy**			
Yes	31	3929 (1710–6278)	0.003
No	27	1300 (56–3807)	
**Anti-diabetic drug**			
Metformin			
Yes	36	3642 (9674–5242)	NS
No	22	1930 (125–3913)	
Sulfonyloureas			
Yes	24	1049 (56–3359)	0.004
No	34	4067 (1464–5912)	
Alpha-glucosidase inhibitors			
Yes	6	3215 (2020–4022)	NS
No	52	2620 (236–4650)	
**Anti-hypertensive drug**			
β-blocker			
Yes	13	4745 (798–6202)	NS
No	45	2575 (259–4341)	
Ca-blockers			
Yes	11	2575 (138–5034)	NS
No	47	3182 (458–4572)	
ACE inhibitor			
Yes	29	1622 (89–4136)	0.029
No	29	3607 (1244–5109)	
Diuretics			
Yes	12	2575 (538–3551)	NS
No	46	3521 (275–4682)	

^a^ Median with the 25th and 75th centiles–quartiles are presented. Abbreviations: M represents male; F represents female; NS represents not significant.

**Table 3:** Correlation between endothelin-1 (*ET-1*) mRNA levels and clinical features/ biochemical markers in patients with type 2 diabetus mellitus (DM)

***ET–1* mRNA (copies/μg total RNA)**	**r–value**	**p-value**
Age (year)	0.067	NS
BMI (kg/m^2^)	−0.149	NS
Length of clinical course (year)	0.292*	0.02
Length of insulin therapy (year)	0.003	NS
**Glycemia	−0.196	NS
**Serum creatinine	0.368*	0.01
**Total cholesterol	−0.249	NS
**Triglycerides	0.127	NS
**HDL cholesterol	−0.004	NS
**LDL cholesterol	−0.447	NS
Glycosylated hemoglobin (%)	−0.014	NS

Statistical significance: *p<0.05; ** mg%, NS represents not significant.

No statistically significant correlation between *ET-1* level and age, gender, bodyweight and height, body mass index, or medication with metformin, α-glucosidase inhibitors, β blockers, calcium blockers, or diuretics was found. There was also no significant correlation between frequent follow-ups, duration of insulin therapy, and smoking habit, and blood levels of glucose, triglycerides, total cholesterol, high-density lipoprotein, low-density lipoproteins, or glycosylated hemoglobin.

## Discussion

Vascular complications resulting from chronic hyperglycemia and insulin resistance are inseparably associated with the pathogenesis of type 2 DM, leading, in turn, to increased mortality rates. The destructive influence of hyperglycemia on eye tissues is manifested in DR. Thus, understanding the pathogenesis of vascular complications in type 2 DM is key for eventually reducing these deleterious sequelae [17]. Changes in the activity of the ET system are significant in initiating the structural and functional damages characteristic of the complications in type 2 DM. In many previously published studies [4–6,17–20], attempts to evaluate the ET-1 concentration in patients with DR as well as the potential correlation between ET-1 concentration and degree of disease progression were made. Increases in ET-1 protein levels in the vitreous were highly correlated with increased concentration of ET-1 in the blood serum of patients with PDR [4]. Moreover, an increase of ET-1 in the vitreous body was observed, together with an increased level of plasma proteins, suggesting an influx of ET-1 from the plasma into the eye [4]. On the other hand, ET-1 may be produced locally by ocular tissues. However, there is a complete lack of data regarding the *ET-1* gene mRNA level, and thus the source of this protein in DR is still unclear. PBMCs, as a source of the ET-1 protein, may significantly influence the molecular changes leading to microangiopathy in DM, independent of the organ studied, including retina.

Our observations suggest that increases in *ET-1* expression in PBMCs are associated with development of microangiopathy, which complicates the course of type 2 DM. Given stimulation of proliferation and migration of endothelial cells by ET-1, a hypothesis may be formulated that increased expression of *ET-1* in PBMCs is involved in initiation of the molecular changes leading to microangiopathy. These observations are in line with those of other studies investigating the level of plasma ET-1 in venous blood of patients with type 2 DM complicated by DR [5,6,17–20]. However, our results were obtained for the whole population of PBMCs. Thus we cannot indicate which particular cellular population is the main source of ET-1.This information would be very helpful in understanding the basis of molecular changes leading to diabetic retinopathy.

Our results suggest relationships between *ET-1* expression and such factors as duration of clinical course of disease, insulin therapy, medication with sulfonylureas or ACE inhibitors, and serum creatinine levels. Prolonged duration of DM results in an increased mRNA level of *ET-1*, with subsequent increase of hyperglycemic incidents that lead to increased activity of kinase C protein [21,22], production of insoluble polymers (so-called advanced glycation endproducts) [2], and increased synthesis of diacyloglycerol [21,23–25]. All of the above result in increased generation of free radicals, which in turn activate proliferative changes and induce hyperglycemia-related injury to tissues, blood vessels, and the endothelium.

Another factor associated with *ET-1* expression in patients with type 2 DM is insulin therapy. The increase in expression of *ET-1* in patients receiving insulin is consistent with results of experimental studies of effects of insulin administration on ET-1 plasma levels under physiologic conditions [26]. Both Ferri et al. [27] and Piatti et al. [28] proved that there is a correlation between plasma levels of ET-1 and dosage (and timing) of insulin administered to euglycemic patients with type 2 DM and hyperinsulinemia. The relationship between plasma levels of ET-1 and insulin (both endogenous and exogenous) is suggested by the fact that plasma levels of ET-1 are higher in patients with type 2 DM than in those with type 1 DM [19], most likely due to higher insulin levels and associated insulin resistance. It is also conceivable that endogenous and exogenous insulin may affect expression of *ET-1*.

The next factor found to be associated with expression of *ET-1* is medication with the sulfonylureas. In our study statistically significant difference in expression of *ET-1* between patients treated with sulfonylurea and those that are not was revealed. The lack of a difference in *ET-1* mRNA levels between patients with DM without DR and the control population would be caused by therapy with sulfonylureas. The *ET-1* mRNA level may be modulated by this agent, which suggests the potential protective effect of such therapy in DM. The potential pharmaceutical modulation of ET-1 release was previously described by Vingolo et al. [29]. They had shown that Defibrotide had multi-site activity, which reduced or delayed the need for laser treatment in NPDR patients. However, there are no data regarding the molecular mechanism by which the sulfonylureas can cause a decrease in *ET-1* expression.

Medication with ACE inhibitors is another factor associated with reduction in expression of *ET-1*. Our statistical analysis revealed that the level of *ET-1* mRNA is definitely lower in patients treated with ACE inhibitors. This suggests that activation of the rennin–angiotensin–aldosterone system plays an important role in the pathogenesis of both microangiopathy and macroangiopathy in DM patients [30]. Inhibition of the rennin–angiotensin–aldosterone system may be achieved by administration of ACE inhibitors. These therapeutic agents delay not only development and progression of vascular pathology, e.g., nephropathy, but probably also retinopathy in patients with type 1 or 2 DM.

Serum level of creatinine is yet another factor influencing expression of *ET-1*. Statistical analysis of *ET-1* expression in patients with DR complicating type 2 DM revealed a positive correlation between increased *ET-1* mRNA level and serum levels of creatinine. However, all currently studied patients had normal serum creatinine levels and had negative albumin strip test results. This suggests that the *ET-1* mRNA level is a more sensitive marker of early epithelial damage within glomeruli, complicating the course of type 2 DM. Results of animal studies support this hypothesis [31,32].

Our analyses suggest that the *ET-1* mRNA level in PBMCs is associated with severity of DR in patients with type 2 DM. A long-standing clinical course of DM; medication with insulin, sulfonylurea, or ACE inhibitors; and serum creatinine levels are factors associated with changes in *ET-1* expression. There is, however, a need to study a larger population to confirm the usefulness of quantitative determination of *ET-1* mRNA in PBMCs for monitoring progression of retinal changes in patients with type 2 DM.
